# Sleep problems in Parkinson's disease: a community-based study in Norway

**DOI:** 10.1186/1471-2377-12-71

**Published:** 2012-08-10

**Authors:** Elisabeth Svensson, Antoine G Beiske, Jon Håvard Loge, Kornelia K Beiske, Børge Sivertsen

**Affiliations:** 1 Department of Adult Mental Health, Division of Mental Health, Norwegian Institute of Public Health, Oslo, Norway; 2 Department of Neurology, Akershus University Hospital, Akershus, Norway; 3 National Resource Centre for Late Effects after Cancer Treatment, Oslo University Hospital, Oslo, Norway; 4 Department of Behavioural Sciences, University of Oslo, Oslo, Norway; 5 Department of Clinical Psychology, University of Bergen, Bergen, Norway; 6 Division of Psychiatry, Helse Fonna HF, Haugesund, Norway

**Keywords:** Sleep, Parkinson, Prevalence, Norway, Associated factors

## Abstract

**Background:**

The purpose of this study was to examine the prevalence of sleep problems in a community-based sample of patients with Parkinson’s disease (PD) in Norway, and their associated factors.

**Methods:**

176 consecutive PD outpatients (41% females) were included in a study of non-motor symptoms, including sleep problems. All participants responded to the Parkinson’s Disease Sleep Scale (PDSS), where an overall score below 82 or a score below 5 on a sub-item indicate possible sleep problem. Factors associated with sleep were also investigated, with special emphasis on severity of PD, fatigue, mental health and restless legs syndrome (RLS).

**Results:**

The mean age was 68.5 years (range 35–90); the mean Hoehn and Yahr stage was 2.11 (SD 0.86), and the mean UPDRS part III was 22.3 (SD 11.7). Sleep problems were common among PD patients. While only 17% of the sample had an overall score below 82 on the PDSS, 70% of the patients had a score below 5 on one item. There was no significant association between PD severity and any of the sleep items in the PDSS; whereas fatigue, mental health problems, and RLS were associated with PDSS score.

**Conclusions:**

The current findings call for increased awareness of sleep problems in PD patients, especially focusing on the association with mental health problems, fatigue and RLS.

## Background

Parkinson’s Disease (PD) is the second most common neurodegenerative disorder, and primarily affects the elderly: while approximately 1% of people aged 65 to 69 suffer from PD, the prevalence increases to nearly 5% among people aged 80 to 84 [1]. PD is characterized not only by movement abnormalities, but also by non-motor symptoms such as fatigue, pain, and sleep problems [2-4]. Sleep problems are common; community studies have reported that about 60% of PD patients had a sleep disorder compared to 33% of the controls [5]. Common sleep problems include insomnia symptoms (difficulty initiating and maintaining sleep), excessive daytime somnolence, and REM sleep behaviour disorder (RBD)[6]. However, even though this is a common problem, the diagnostic accuracy of sleep problems in PD patients is only 60% [7].

It has been recommended that assessment of PD should also include a disease-specific instrument for measurement of sleep disturbances [8]. The Parkinson’s Disease Sleep Scale (PDSS) was developed on this background and also because the generic sleep assessment scales do not systematically address and quantify the most common sleep problems in PD patients [9]. As such, this bedside tool can provide an indication for treatment of the sleep problems commonly accompanying PD.

Sleep and its associated factors in PD patients have been examined in several studies [6,10], but the predictive factors are found to vary and the results must be considered contradictory. One example is the relationship between sleep disturbance and fatigue: although it has been shown that sleep problems and fatigue frequently co-occur in PD, it has also been suggested that they should be regarded as distinct symptoms that must be understood and managed separately [11]. Another and more consistent relationship is that between depression and sleep problems in PD [12-14]. Symptoms of restless legs syndrome (RLS) are commonly reported by PD-patients, and such symptoms have also been associated with sleep problems [15]. Finally, there are reports of an association between PD severity and sleep problems [9] however, these results are also inconsistent [5].

The purpose of this study was to examine self-reported sleep problems among PD patients in a community-based sample from Norway. Associated factors with sleep problems were also investigated, with special emphasis on disease stage, age and gender, mental health, fatigue and restless legs syndrome.

## Methods

### Material

All PD-patients living in the eastern part of Akershus county, Norway (320 000 inhabitants), were defined as the target population. Detailed information about the inclusion has been published previously (see [3]). In short, a total of 413 PD patients were identified in the out-patient registry at the Department of Neurology, Akershus University Hospital; the only Neurological Department in the county. Only 243 patients fulfilled the inclusion criteria: PD diagnosis according to published diagnostic criteria [16], as well as mentally (Mini Mental State Examination, with a score ≥ 23 [17]) and physically able to complete interviews, self-report questionnaires and clinical examinations lasting for at least two hours. The data collection was organised as part of a yearly follow-up consultation. If patients did not want to participate, the regular consultations were conducted as scheduled. Due to financial restraints the inclusion period had to be shortened before the entire cohort was examined. The last forty-one eligible patients were therefore not invited to participate. Thus, 202 eligible patients were invited, 17 rejected, six did not answer, and three did not show up. In total, 176 eligible patients were included consecutively until the end of the inclusion-period, yielding a response rate of 87% (176/202). All patients were on dopaminergic medications. In addition, in total 8.5% were on treatment with anti-depressants/anticonvulsants with the intent of anti-depressive or pain-relieving effects [3].

### Instruments

Through a structured interview, conducted as part of the clinical examination, information on PD, such as date of first symptom, date of diagnosis, and date of initiating therapy, were collected and recorded. After the clinical examination, patients were asked to complete a self-report questionnaire, which consisted of, among others, demographic information, and well-established instruments for assessment of sleep disturbances, fatigue, mental health problems and restless legs syndrome.

Staging of the disease was based upon the findings of the clinical examination and in accordance with present standards (Hoehn and Yahr Staging [18] and Unified Parkinson’s Disease Rating Scale, UPDRS [19]). Cognitive functioning was assessed by the Mini Mental State Examination (MMSE) [17]. All patients suffering from motor fluctuations (the fluctuating stage of the disease = on/off-periods) were examined during the on-period.

Sleep problems were assessed by the Parkinson’s disease Sleep Scale (PDSS)[9]. The PDSS include 15 questions addressing the following 8 items: Overall quality of night’s sleep (question 1), sleep onset and maintenance insomnia (questions 2 and 3), nocturnal restlessness (questions 4 and 5), nocturnal psychosis (questions 6 and 7), nocturia (questions 8 and 9), nocturnal motor symptoms (questions 10–13), sleep refreshment (question 14) and daytime dozing (question 15). The response format is a 0–10 cm visual analogue scale, where 0 indicates that a symptom *is severe and always experienced*, while 10 means *being free of symptoms*. The score on each item is summarised into a total score giving a range of scores from 0 (most severe) to 150 (free of symptoms). Based on comparisons with polysomnography and other sleep questionnaires such as the Epworth Sleep scale, the PDSS appears to be a reliable tool to evaluate sleep characteristics in PD patients. [20] It is suggested that individuals obtaining an overall score below 82 or a score under 5 on any sub- item on the PDSS should be referred for formal sleep studies [21,22].

Fatigue was assessed by the Norwegian version of the Fatigue Questionnaire FQ [23,24]. The FQ includes 11 items, and previous factor analyses support a two-factor solution; mental and physical fatigue. The sum score of responses to all 11 items is designated total fatigue, with minimum/maximum scores of 0/33. The FQ was originally validated in primary care, and has demonstrated good face- and discriminant validity as well as good and stable psychometric properties across populations [23]. Reference data on fatigue levels from the Norwegian general population exists [24].

Mental health was assessed by the mental health (MH) subscale (5 items) of the Short Form 36 (SF-36) [25]. The SF-36 has been validated in the general Norwegian population [26]. Each item of the mental health subscale is scored on a 6 point category scale. The scores on all items are summed and transformed into a 0–100 scale (0 = poorest possible health state, 100 = best possible health state). A score below 50 is thought to indicate poor mental health.

Restless legs syndrome was assessed in a clinical interview by an experienced neurologist, based on the 4 essential criteria put forward by the International Restless Legs Syndrome Study Group[27], resulting in a dichotomous score (no/yes).

The study was approved by the Regional Committee for Medical Research Ethics. Appropriate informed consent was obtained from all respondents.

### Statistical methods

Descriptive statistics for sleep problems, clinical and demographic variables were calculated. Further characteristics of PDSS were investigated, such as proportion of patients scoring under 82 points. The entire UPDRS was conducted, but only results from stage III will be reported. To analyse possible associations between sleep problems and characteristics of PD, demographic status, fatigue, mental health and co-morbidities, linear multiple regression was used. Crude bivariate analyses were conducted (data not shown). A stepwise approach was conducted; block one including demographic factors (age and gender), block two adding disease characteristics (disease duration and UPDRS part III) and block three adding other possible explanatory factors (fatigue, mental health, restless legs syndrome).

A significance level of 0.05 was chosen. Analyses were performed using STATA version 11.

## Results

### Sample characteristics

Of the 176 patients examined, 41% were women, and the mean age was 68.5 years (SD 8.8 years). The mean UPDRS part III was 22.3 (SD11.7). The mean Hoehn and Yahr stage was 2.11 (SD 0.86), the median is 2, and the interquartile range is 1. More specifically, 39.2% are in stage 1, 38.1% are in stage 2, 14.8% are in stage 3 and 8% in stage 4 of Hoehn and Yahr. For patients suffering from motor fluctuations (the fluctuating stage of the disease = on-/off-periods), all were examined during the on-period. The mean duration of PD was 7.5 years (SD 5.3) and the mean MMSE was 28 (range 23–30). The mean mental health SF-36 score was 72.3 (SD 18.8), and the mean total fatigue score was 15 (SD 5.0). RLS was reported by 27% of the patients, and was significantly more common in patients with Hoehn and Yahr stage 1–2 (26%), compared to those with stage 3–4 (10%).

### Sleep problems in PD patients

The overall mean total PDSS score was 102.8 (SD 23.3), and 17% (N = 32) of the patients had a total PDSS score under 82. A more detailed examination of the PDSS sub-scales and proportion of patients scoring below 5 are shown in Table 1). In total, 70% had a total score under 5 on one or more items of the PDSS. There were no significant differences in Hoehn and Yahr stage 1–2 vs 3–4 for any of the PDSS items.

**Table 1:** Sleep problems, by PDSS items, for 176 patients with Parkinson’s disease

	**mean (± SD)**	**% scoring <5**
Item 1: Quality of nights sleep	6.0 (2.9)	37
Item 2: Sleep onset and maintenance insomnia	7.0 (2.5)	25
Item 3: Nocturnal restlessness	5.7 (2.8)	36
Item 4: Nocturnal psychosis	8.2 (2.0)	8
Item 5: Nocturia	5.8 (2.0)	26
Item 6: Nocturnal motor symptoms	7.5 (2.0)	11
Item 7: Sleep refreshment	6.1 (3.1)	35
Item 8: Daytime dozing	7.1 (3.3)	28
Total	102.8 (23.3)	70

Factors associated with sleep problems are shown in Table 2. Only total fatigue score, RLS and mental health problems were factors associated with PDSS score.

**Table 2:** Demographic and clinical factors associated with sleep problems (assessed by total PDSS score) from a block-wise multiple regression analyses in 176 Norwegian PD patients

	**Block one**		**Block two**		**Block three**	
**Variable**	**Beta**	**p-value**	**Beta**	**p-value**	**Beta**	**p-value**
Age (yrs)	0.12	0.1	0.14	0.07	0.01	0.1
Gender	−0.04	0.6	-.05	0.5	0.07	0.2
Disease duration (yrs)			−0.12	0.13	−0.09	0.2
UPDRS sum part III			−0.15	0.07	−0.05	0.5
Mental health (SF-36)					0.36	<0.001
Fatigue (FQ)					−0.17	0.02
Restless legs syndrome					−0.34	<0.001

The adjusted R2 for the final model was 0.39.

## Discussion

Sleep problems were common among the PD patients examined. No association was found between PD severity and sleep problems in this community-based population of medicated patients, of whom 71% had a mild disease. In contrast in this group, the sleep problems were related to mental health problems, fatigue and RLS.

The prevalence of sleep problems among PD patients depends on the population examined and the definition of sleep problems (i.e. the assessment method). In Norway, the prevalence of PD is about 111 per 100.000 inhabitants [28], suggesting about 355 patients in our recruitment area. Thus, our initial population of 345 was probably close to all cases in the catchment area. The response rate was 87%. We therefore consider the present study to have good external validity for a community-based study, although it was conducted at a single institution. On the other hand, although this study might be representative for the Norwegian population, the results are not generalizable to the global PD population at large. For sleep assessment use, the PDSS was used. The clinimetric properties of the instrument are valid and widely used. However, there are some limitations of the instrument, as it does not measure some of the sleep disorders, such as REM behaviours disorder, and others may be underdiagnosed, such as diurnal somnolence.

A limitation of the generalizability is that the study does not address sleep problems in PD patients with dementia or in patients otherwise unable to utilize self-report instruments for data collection. Another limitation of the study is that there is no control population, and since PDSS is a disease-specific scale, there are no Norwegian norm data available. Furthermore, all patients are examined when in medicated state, and we did not further specifically explore the relationship between the PD medications and sleep. The treatment of the disease may affect the sleep disorder; low doses of dopamine agonists has been shown to promote sleep, whereas high doses both during the day and in the evening may lead to increased nocturnal awakenings, reduced slow-wave sleep, and decreased sleep continuity [29].

In the current study, only 17% of the patients had a PDSS score under 82, which is the recommended clinical cut-off for the PDSS. However, when examining the specific PDSS sub-scales, over a third of the sample reported poor quality of sleep, nocturnal restlessness, and poor sleep refreshment. In total, 70% had a total score under 5 on one or more items of the PDSS. Consequently, several of these patients are candidates for referral to further formal sleep tests according to the recommendations for the use of PDSS. The availability of objective sleep studies for verification and established treatment options for sleep problems should prompt physicians to refer PD patients with specific sleep complaints to PSG and sleep apnoea studies. Still, in Norway there is a strikingly low rate of PD-patients referred to the department of neurophysiology at Akershus university hospital, even though the high prevalence of sleep problems in this patient group is widely accepted.

In the group-wise regression model, only three factors were significantly associated with PDSS score, namely poor mental health, restless legs syndrome, and fatigue. Our finding that mental health problems strongly predicted sleep problems in PD patients was not surprising. The close relationship between the two is well known, both in the general population [30] and in patients with PD [31,32]. Although the direction of causality between them is often difficult to establish, a large population-based longitudinal study recently showed that both insomnia and depression strongly and similarly predicted the onset of the other [33]. In PD, however, the extent of these associations and how they relate to the clinical heterogeneity in PD is not fully understood, and there is therefore a need for longitudinal studies to examine the nature of the entwined relationship between sleep problems and depression in this patient group.

In the current study, sleep problems as measured by the PDSS, was significantly associated with fatigue, which is in accordance with other studies [34]. In contrast, studies assessing daytime somnolence (such as the Epworth Sleepiness Scale) rather than nocturnal problems, generally do not find such an association [35,36]. As fatigue is an undifferentiated problem present in the general population and associated with several somatic complaints, the entwined relationship between sleep problems and fatigue needs to be elucidated.

In this study, the prevalence of RLS was 27% and an association was found with sleep problems. Few have examined this association in PD patients, and previous results are contradictory. However, those using a disease specific sleep scale, such as SCOPA-Sleep or PDSS report an association [37,38], while those finding no association tended to use generic sleep assessment scales, such as the Epworth Sleepiness Scale or the Pittsburgh Sleep Quality Index [15,39]. The PDSS has one item on nocturnal restlessness (item 4), and as in other studies those with RLS have significantly lower score on this item [22]. The relationship between PDSS and RLS is still significant when item4 is removed from the analysis (data not shown). In line with earlier suggestions, the PDSS can be helpful in identifying RLS symptoms in PD patients.

An interesting and surprising finding was that stage of disease, measured by both Hoehn and Yahr and UPDRS sum part III separately, was not associated with PDSS score in this population. This finding is in disagreement with other studies examining the association between sleep problems and PD severity [9,22]. However, in this community-based study of medicated patients, 71% had a mild disease. We would have expected that patients with severe PD-symptoms also reported more sleep problems than patients with less severe PD. It should be noted that the current study did not include the most severe PD patients (Hoehn and Yahr stage 5), and as such, it might be that sleep problems are more prevalent among the most severely affected PD patients.

## Conclusion

The current findings call for increased awareness of sleep problems in PD patients, especially focusing on the association with mental health problems, fatigue and RLS.

## Competing interest

None of the authors have competing interests.

## Authors’ contribution

AGB and JHL participated in the conception, organization and execution of the data collection. ES and BS had the idea for the study, and performed the statistical analyses and wrote the first draft, while AGB, JHL and KKB participated in review and critique of the manuscript. All authors read and approved the final manuscript.

## Authors’ roles

1. Research project: A. Conception, B. Organization, C. Execution

2. Statistical Analysis: A. Design, B. Execution, C. Review and Critique

3. Manuscript: A. Writing of the first draft, B. Review and Critique

Svensson: 2A, 2B, 3A, 3B

AG Beiske 1A, 1B, 1C, 2C, 3A, 3B

Loge 1A, 2A, 2C, 3B

KK Beiske 2C, 3B

Sivertsen 2A, 2B, 3A, 3B

## Financial support

The work was supported by a grant from Ullevål University Hospital Oslo, Norway which made the carrying out of the study possible.

## Pre-publication history

The pre-publication history for this paper can be accessed here:

http://www.biomedcentral.com/1471-2377/12/71/prepub
